# DNA Hypomethylation, Ambient Particulate Matter, and Increased Blood Pressure: Findings From Controlled Human Exposure Experiments

**DOI:** 10.1161/JAHA.113.000212

**Published:** 2013-06-21

**Authors:** Andrea Bellavia, Bruce Urch, Mary Speck, Robert D. Brook, Jeremy A. Scott, Benedetta Albetti, Behrooz Behbod, Michelle North, Linda Valeri, Pier Alberto Bertazzi, Frances Silverman, Diane Gold, Andrea A. Baccarelli

**Affiliations:** 1 Department of Environmental Health, Harvard School of Public Health, Boston, MA (A.B., B.B., D.G., A.B.); 2 Gage Occupational and Environmental Health Unit, St. Michael's Hospital, Toronto, Ontario, Canada (B.U., M.S., J.A.S., M.N., F.S.); 3 Division of Occupational and Environmental Health, Dalla Lana School of Public Health, University of Toronto, Toronto, Ontario, Canada (B.U., M.S., J.A.S., M.N., F.S.); 4 Division of Cardiovascular Medicine, University of Michigan, Ann Arbor, MI (R.D.B.); 5 Department of Biostatistics, Harvard School of Public Health, Boston, MA (L.V.); 6 Department of Occupational and Environmental Health, Universita' degli Studi di Milano and Fondazione Ca' Granda Policlinico, Milan, Italy (B.A., P.A.B.); 7 Channing Laboratory, Brigham and Women's Hospital, Harvard Medical School, Boston, MA (D.G.)

**Keywords:** air pollution, blood pressure, DNA methylation, epigenetics, mediation

## Abstract

**Background:**

Short‐term exposures to fine (<2.5 μm aerodynamic diameter) ambient particulate‐matter (PM) have been related with increased blood pressure (BP) in controlled‐human exposure and community‐based studies. However, whether coarse (2.5 to 10 μm) PM exposure increases BP is uncertain. Recent observational studies have linked PM exposures with blood DNA hypomethylation, an epigenetic alteration that activates inflammatory and vascular responses. No experimental evidence is available to confirm those observational data and demonstrate the relations between PM, hypomethylation, and BP.

**Methods and Results:**

We conducted a cross‐over trial of controlled‐human exposure to concentrated ambient particles (CAPs). Fifteen healthy adult participants were exposed for 130 minutes to fine CAPs, coarse CAPs, or HEPA‐filtered medical air (control) in randomized order with ≥2‐week washout. Repetitive‐element (Alu, long interspersed nuclear element‐1 [LINE‐1]) and candidate‐gene (TLR4, IL‐12, IL‐6, iNOS) blood methylation, systolic and diastolic BP were measured pre‐ and postexposure. After adjustment for multiple comparisons, fine CAPs exposure lowered Alu methylation (β‐standardized=−0.74, adjusted‐*P*=0.03); coarse CAPs exposure lowered TLR4 methylation (β‐standardized=−0.27, adjusted‐*P*=0.04). Both fine and coarse CAPs determined significantly increased systolic BP (β=2.53 mm Hg, *P*=0.001; β=1.56 mm Hg, *P*=0.03, respectively) and nonsignificantly increased diastolic BP (β=0.98 mm Hg, *P*=0.12; β=0.82 mm Hg, *P*=0.11, respectively). Decreased Alu and TLR4 methylation was associated with higher postexposure DBP (β‐standardized=0.41, *P*=0.04; and β‐standardized=0.84, *P*=0.02; respectively). Decreased TLR4 methylation was associated with higher postexposure SBP (β‐standardized=1.45, *P*=0.01).

**Conclusions:**

Our findings provide novel evidence of effects of coarse PM on BP and confirm effects of fine PM. Our results provide the first experimental evidence of PM‐induced DNA hypomethylation and its correlation to BP.

## Introduction

Air pollution is a major public health concern in the United States and worldwide, accounting for approximately 800 000 deaths annually.^1^ Historical episodes of accumulations of ambient particulate matter (PM), such as in London in December 1952 or in Donora Valley in the last 3 days of October 1948, have been associated with up to >10 times increased death rates, mostly from cardiovascular disease (CVD).^2^ In the past 30 years, massive efforts in controlling emissions have led to substantial lowering of air pollution levels. However, in the presence of stagnating weather, peaks more than 10 times higher than the background levels of PM pollution are still frequently recorded in U.S. cities^3^ and followed by increased CVD hospitalization and mortality within hours or days.^4–5^ A recent comparative study showed that PM and traffic pollution exposures, because they expose millions to unhealthy air, are among the most frequent triggers of myocardial infarction at the population level, with about twice as many events as heavy alcohol consumption and >10 times than cocaine abuse.^6^ As recently as in July 2012, warnings for unhealthy PM levels were issued for most large U.S. cities, as reported by the U.S. Environmental Protection Agency.^3^

Blood pressure (BP) can change rapidly in response to environmental stressors^7–8^ and has been proposed as a primary intermediate response for acute PM‐related cardiovascular events.^4,7^ Airborne PM ≤2.5 μm (PM_2.5_ or fine particles) in aerodynamic diameter, as well as larger particles between 2.5 and 10 μm (coarse particles), can be inhaled and deposited in the upper and lower airways. Because of their smaller size, fine particles can reach more deeply into the lungs and have been suggested to have more harmful effects on cardiovascular outcomes.^4^ Observational data and previous studies of controlled human exposures have reproducibly shown rapid adverse effects on BP as early as 2 hours during PM exposure.^7,9^ An increase as small as 1 mm Hg in usual systolic BP is estimated to increase the risk of CVD deaths by 2% to 4%,^10–11^ and transient increases have been linked to PM‐related triggering of cardiovascular events.^4,12^ The limited understanding of the mechanisms linking air pollution exposure to cardiovascular outcomes, including effects on BP, is identified as a critical research and clinical gap in a 2010 statement of the American Heart Association.^4^

DNA methylation, the most studied of the epigenetic mechanisms, is a natural process that suppresses gene expression via addition of methyl groups to the DNA. Loss of methylation in inflammatory genes has been shown in lymphocytes as early as 20 minutes after antigen presentation.^13^ Findings from human observational studies have suggested that PM exposure may determine loss of methylation in blood DNA, potentially reflecting activation of proinflammatory states in blood leukocytes.^14^ Specifically, PM‐related hypomethylation has been repeatedly observed in transposable repeated elements, including *Alu*^15–17^ and long interspersed nuclear element‐1 (LINE‐1),^15–18^ as well as in candidate proinflammatory genes.^16^ Consistent with these observations, reduced methylation of genomic DNA in blood has also been observed in patients with cardiovascular disease,^14,19^ or CVD‐related conditions and risk factors, including atherosclerosis,^20^ oxidative stress,^21^ and aging.^22^ However, current evidence on PM‐induced hypomethylation rests entirely on observational studies, and no human experimental data are yet available to demonstrate effects of air pollution on DNA methylation. In addition, whether PM‐induced hypomethylation mediates the effects of PM on cardiovascular outcomes, such as those on BP, has never been tested.

Controlled studies on human‐exposure to concentrated ambient particles (CAPs) provide a unique opportunity to simulate air pollution peaks, while allowing for experimental control of the exposures. Previous controlled human exposure experiments have shown increased BP after exposure to fine CAPs^9,23^—consistent with observational studies that have associated short‐term PM_2.5_ exposure with BP.^4^ To the best of our knowledge, changes in BP after coarse CAP exposure have not yet been documented. Albeit mostly deposited and cleared in the upper airways, coarse particles are enriched in organic components that activate innate inflammatory responses.^24^ Activation of specific innate immune responses in circulating leukocytes, such as those mediated through increased expression of the toll‐like receptor‐4 (TLR4), have been linked with BP and hypertension.^25^ Herein, we report the results of a double‐blind randomized cross‐over trial of controlled human exposures to fine and coarse CAPs. We experimentally determined effects on blood DNA methylation of LINE‐1 and *Alu* repetitive elements and candidate proinflammatory genes (*TLR4*,* IL‐12*,* IL‐6*,* iNOS*). In addition to evaluating CAPs effects on blood DNA methylation, we tested for CAPs‐induced effects on BP and conducted mediation analysis to estimate the proportion of the effects on BP mediated by DNA methylation.

## Materials and Methods

### Study Participants

The study included 15 healthy volunteers between 18 and 60 years of age. All participants were nonsmokers and free of CVD. All experiments were conducted between January 2008–March 2010 at the Gage Occupational and Environmental Health Unit, University of Toronto, Ontario, Canada. Exclusion criteria included a fasting total cholesterol >6.2 mmol/L, glucose >7 mmol/L, hypertension (resting BP >140/90 mm Hg), pregnancy/lactation, or ECG abnormalities. The study received institutional review board approval from St. Michael's Hospital and University of Toronto. All participants provided written informed consent before enrolling.

### Study Design and Exposure Protocol

Using a double‐blind randomized placebo‐controlled cross‐over design, each participant underwent 3 exposures in random order: (i) fine CAPs; (ii) coarse CAPs; and (iii) High‐Efficiency‐Particulate‐Air (HEPA)‐filtered medical air (control), separated by a minimum 2‐week washout period. Volunteers and study personnel were blinded to the exposure order. Only the technologist who generated the exposure was aware of the exposure type. We utilized a controlled human exposure facility that concentrates fine or coarse particles under controlled conditions, using high‐flow (5000 L/min) Harvard ambient particle concentrators (see details in Data S1). Briefly, ambient particles were drawn into a 1.8 m high PM_10_ inlet located 10 m from a busy 4‐lane downtown Toronto street, with ≈2500 vehicles passing during the 130‐minute exposure. The CAP airstream was delivered directly to the volunteer who was seated inside a Lexan and steel tube frame enclosure (4.9 m^3^, see exposure apparatus during one of the experiments in Figure S1). Participants were at rest and breathing freely (no mouthpiece) via an “oxygen type” facemask covering their nose and mouth.^26^ The target levels for the fine and coarse CAP experiments were 250 and 200 μg/m^3^, respectively. The CAP/filtered air delivery system was designed so that there were no visual cues as to the exposure type while participants were seated in the exposure chamber.

### BP Measures and Blood Sample Collection

All participants fasted (>8 hours) prior to their arrival at the facility at ≈7:30 am. Blood samples were collected at ≈9 am. Afterward, volunteers underwent the 130‐minute exposure, at rest. Seated BP was obtained 10 minutes before exposure and 5 minutes after completion of the exposure. Postexposure blood samples were collected ≈1 hour after the end of the exposure. A standardized protocol for BP measurements was used as recommended by the American Heart Association (see Data S1, Figure S2).^27^

### DNA Methylation Analyses

Buffy coat was immediately obtained from blood in a preprocessing laboratory adjacent to the exposure facility, aliquoted, and frozen at −20°C until DNA isolation. All laboratory procedures on the buffy‐coat samples, from DNA isolation through DNA methylation analyses, were performed in a single batch. DNA was purified using Qiagen DNeasy Blood and Tissue Kit (Qiagen). All samples from each participant were placed on the same analytical plate to avoid plate effects. DNA methylation analyses were performed by bisulfite PCR‐Pyrosequencing. We performed DNA methylation analyses of *Alu* and LINE‐1 repeated sequences, as described previously,^16^ which allows for the amplification of a representative pool of repetitive elements. We developed assays for *TLR4*,* IL6*,* IL12*, and *iNOS* methylation by locating their promoters using the Genomatix Software (Genomatix Software Inc) and amplified the sequences shown in Tables S1 and S2. In each assay, we measured %5mC at multiple CpG dinucleotides (Table S1). Every sample was tested in duplicate to confirm reliability.

### Statistical Analysis

In each blood sample, DNA methylation analysis produced 8 values each for LINE‐1 and *TLR4* (methylation at 4 CpGs replicated in 2 runs), 6 values for *Alu* (methylation at 3 CpGs replicated in 2 runs), and 4 values each for *IL‐6*,* IL‐12*, and *iNOS* (methylation at 2 CpGs replicated in 2 runs). In addition, methylation was measured in each participant at 2 time points (pre‐ and postexposure) in each of the 3 randomized experiments. To account for this data structure and consider within‐individual effects, we used mixed‐effect models (PROC MIXED in SAS V9.2).^16^ We fitted mixed‐effect models with a random intercept for each subject—which captures the correlation among measurements within the same subject; a random intercept for each CpG—which captures the correlation among measurements within the same CpG position; and a random slope for each position—which captures potential different effects of the exposures across the different positions. To control for potential confounding due to period effect, we also included a numeric variable indicating the order of exposure.

For each DNA methylation marker we assumed the following:
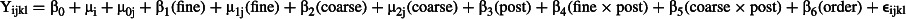
1where Y_ijkl_ is the value of methylation in subject i, CpG position j, time k (pre‐ or postexpsosure) and experiment l (fine CAPs, coarse CAPs, or medical air); β_0_ is the overall intercept, which indicates the average methylation in the control group (medical air) in preexposure samples; μ_i_ is the random intercept for the subject i; μ_0j_ is the random intercept for each position; β_1_ and β_2_ are the main effects of the exposure to fine CAPs and to coarse CAPs, respectively, compared to the control exposure; β_3_ is the main effect of time (postexposure compared to preexposure); μ_1j_ and μ_2j_ are the random slopes of the different CpG positions for each exposure; β_4_ and β_5_ are the interaction effects between exposure (fine and coarse, respectively) and time; β_6_ represents the period effect.

The choice of the 6 methylation markers, and of BP as the clinical outcome, was made a priori. No other methylation markers or outcomes were examined. *P* values from the model described in equation 1 were adjusted for multiple comparisons using a permutation test that accounts for data correlation.^28^ Methylation data are expected to be correlated and statistical tests are likely to be not independent. In this situation, commonly used methods of multiple‐testing correction such as Bonferroni and FDR will overestimate the adjusted *P* value. Permutation test represents a straightforward—albeit computationally heavier—and accurate approach to correct for multiple comparisons.^28^ Briefly, we randomly permuted the exposures within subject and then regressed each of the 6 DNA methylation markers over the exposures on this permuted dataset. The permutation breaks the link in the data between the exposures and DNA methylation, thus the dataset generated will be under the null hypothesis. We repeated this process 1000 times. A total of 12 000 *P* values (6 genes×1000 datasets×2 exposures) were obtained. The adjusted permutation *P* value was equal to the number of simulations with *P* value smaller than the observed *P* value divided by 12 000. Adjusted permutation *P* values <0.05 were considered significant and reported alongside the nominal 95% CIs and *P* values.

We then examined the effects of the exposures on systolic or diastolic BP by using the following mixed‐effect models:
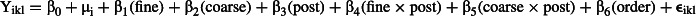
2where Y_ikl_ is the measured value of either systolic or diastolic BP for subject i, time k, and experiment l; β_0_ is the overall intercept, which indicates the average value of BP in the control group (medical air) preexposure; β_1_ and β_2_ are the main effects of the exposure, respectively, to fine CAP and to coarse CAP compared to the control exposure; μ_i_ is the random intercept for the subject i; β_3_ is the main effect of time (postexposure compared to preexposure); β_4_ and β_5_ are the interaction effects between exposure (fine and coarse, respectively) and time; β_6_ represents the period effect.

From the regression coefficients of the models in equations 1 or 2, group means can be derived as the average values of the dependent variables for each combination of exposure and time. In our primary analysis, we present within‐subject mean differences between postexposure measurements (ie, fine versus medical air; and coarse versus medical air). For those outcomes showing a statistically significant difference, we are also reporting differences between pre‐ and postexposure means.

We finally evaluated the association of the DNA methylation markers with systolic or diastolic BP levels in postexposure measures. To reduce multiple testing, we prioritized and present in the paper only the results for the methylation markers that showed significant differences after either fine or coarse CAP exposure. For both systolic and diastolic BP, we assumed the following model:
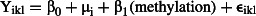
3where Y_ikl_ is the measured value of either systolic and diastolic BP for subject i, time k, and experiment l; β_0_ is the overall intercept; β_1_ is the regression coefficient for each the DNA methylation fitted as the mean of CpG positions and runs; μ_i_ is the random intercept for the subject. A nominal *P* value <0.05 was considered statistically significant.

### Mediation Analysis

We performed mediation analysis to estimate the proportion of the exposure effects on BP mediated by DNA methylation. Based on the a priori assumption that a mediated effect is biologically plausible, this approach decomposes the total observed effect of exposure on BP into a direct effect of exposure and an indirect effect of exposure that acts via the mediator of interest^29–30^ (ie, DNA methylation). Mediation analysis usually requires a significant relation of the outcome to the exposure, a significant relation of the outcome to the mediator and a significant relation of the mediator to the exposure;^31^ as potential mediators, we therefore analyzed the methylation markers that satisfied all these assumptions. In order to establish mediation, a significant relation of the mediator to the outcome, when both the mediator and the exposure are predictors, is also required. To test the latter assumption we evaluated the potential mediators in the following model:
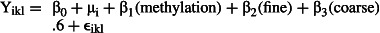
4where Y_ikl_ is the measured value of BP (either systolic or diastolic) for subject i, time k, and experiment l; β_0_ is the overall intercept; μ_i_ is the random intercept for the subject; β_1_ is the regression coefficient for each CpG position; β_2_ and β_3_ are the main effects of the exposure to fine and coarse CAPs, respectively, relative to the medical air control exposure.

Once all these assumptions are verified, it is possible to evaluate the indirect effect, which estimates the size of the effect of CAPs exposure on BP that is mediated by DNA methylation.^32^ We estimated the indirect effect via a mixed‐effect mediation model using PROC MIXED in SAS 9.2.^29–30^

## Results

### Effects of Controlled Exposures on DNA Methylation

Table 1 shows the baseline characteristics of the study participants. The study included 8 male and 7 female participants (average age 27.7 years). The actual average levels of PM mass concentrations achieved during the experiments were 241.8 μg/m^3^, 210.6 μg/m^3^, and 0.6 μg/m^3^ during the fine CAP, coarse CAP, and control exposures, respectively.

**Table 1.** Characteristics of the Study Participants at the Enrollment Visit*, n=15

Characteristics	Mean±SD or n (%)
Age, y	27.7±8.6
Gender	
Male	8 (53.3)
Female	7 (46.7)
Race	
White	10 (66.7)
Black	1 (6.7)
Asian	4 (26.6)
BMI, kg/m^2^	23.2±2.4
Fasting glucose, mmol/L	4.8±0.5
Fasting cholesterol, mmol/L	4.2±0.7
Heart rate, beats/min	67.9±12.6
Systolic BP, mm Hg	117.6±14.1
Diastolic BP, mm Hg	69.1±12.2
White blood cell count, 109/L	5.5±1.1
DNA methylation, % 5mC	
*Alu*	24.2±0.5
LINE‐1	84.3±0.7
*TLR4*	3.6±0.8
*IL‐6*	45.5±7.4
*IL‐12*	94.9±0.7
*iNOS*	62.9±2.8

BMI indicates body mass index; BP, blood pressure; LINE‐1, long interspersed nuclear element‐1; *TLR4*, toll‐like receptor‐4; *IL‐6*, interleukin 6; *IL‐12*, interleukin 12; *iNOS*, inducible nitric oxide synthase gene.

* Variables assessed at a preliminary screening visit conducted before the beginning of the experiments except DNA methylation—methylation values were measured on blood samples collected before the first exposure experiments.

**Figure 1.**
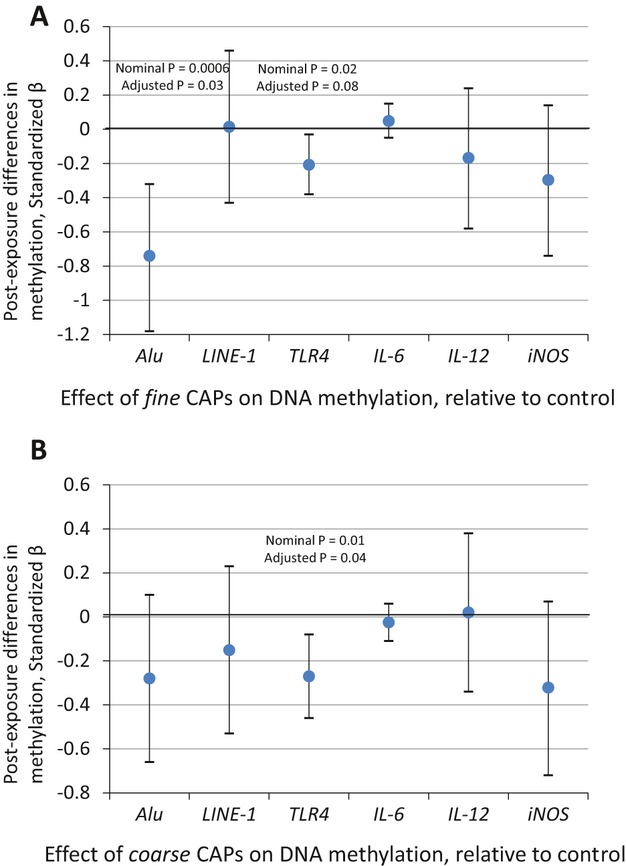
Effect of controlled exposures to fine CAPs (A) and coarse CAPs (B) on blood DNA methylation. Differences in CAPs vs medical air (control) exposures of DNA methylation in postexposure samples reported as standardized βs and 95% confidence intervals. βs indicate the differences between CAPs exposure and control exposure (HEPA‐filtered medical air) in the postexposure blood samples. βs are standardized to express the effect of CAPs exposure on DNA methylation as a fraction of the standard deviation of DNA methylation. Nominal *P* values (*P*), as well as *P* values adjusted for multiple testing (adjusted *P*) are shown for the significant effects. CAP indicates concentrated ambient particle; HEPA, high‐efficiency particulate air; LINE‐1, long interspersed nuclear element‐1; TLR4, toll‐like receptor‐4; IL‐6, interleukin 6; IL‐12, interleukin 12; iNOS, inducible nitric oxide synthase gene.

Figure 1 shows the average postexposure differences in methylation in blood samples collected after exposures to CAPs (fine or coarse) relative to the medical air control exposure, taken as reference (ie, mean within‐subject differences between postexposure measurements obtained from model [1]); because methylation showed large between‐marker differences in mean and range of values, to allow for comparability we report standardized βs expressing the difference between exposures as a fraction of the SD of DNA methylation. *Alu* methylation was significantly lower after fine‐CAPs exposure (standardized β=‐0.74, *P*=0.0006), compared to postmedical air (control) exposure. *TLR4* methylation was significantly lower after both fine‐ (standardized β=‐0.21, *P*=0.02) and coarse CAPs (standardized β=‐0.27, *P*=0.01) exposures, relative to the postcontrol exposure (Figure 1). After *P* value adjustment for multiple comparisons, the postexposure difference in *Alu* methylation between fine CAPs and medical filtered air remained significant (permutation‐adjusted *P*=0.03). The effect on *TLR4* methylation remained significant for coarse CAPs (permutation‐adjusted *P*=0.04), but not for fine CAPs (permutation‐adjusted *P*=0.08). No significant differences were observed in postexposure LINE‐1, *IL‐6*,* IL‐12*, or *iNOS* methylation. Postexposure comparisons for each of the 6 methylation markers are reported in Table S5.

We confirmed these findings by evaluating the average within‐participant change in DNA methylation in postexposure relative to preexposure blood samples in each of the 3 exposures (fine CAPs, coarse CAPs, or control). *Alu* methylation showed a significant average within‐participant decrease in postexposure samples after fine CAPs exposure (standardized β=−0.40, *P*=0.05), and no change after coarse CAPs (standardized β=0.02, *P*=0.86) or control exposures (standardized β=0.20, *P*=0.26). *TLR4* methylation showed a significant average within‐participant decrease after fine (standardized β=−0.21, *P*=0.02) and coarse (standardized β=−0.16, *P*=0.05) CAPs exposures, and no significant changes after control exposure (standardized β=0.11, *P*=0.28).

### Effects of Controlled Exposures on BP

**Figure 2.**
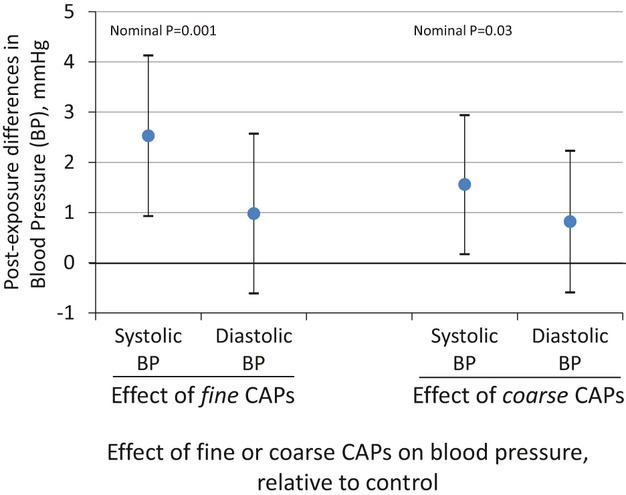
Effect of controlled exposures to fine concentrated ambient particles (CAPs) and coarse CAPs on systolic and diastolic blood pressure (BP). Differences in CAPs vs medical air (control) exposures of systolic and diastolic BP in postexposure measurements. βs and 95% confidence intervals expressing the difference in BP (mm Hg) between CAPs exposures and control exposure (HEPA‐filtered medical air) in postexposures measurements. HEPA indicates high‐efficiency particulate air.

Figure 2 shows the average BP differences after exposures to CAPs (fine or coarse) relative to the control exposure, taken as reference (ie, mean within‐subject differences between postexposure measurements obtained from model [2]);. Systolic BP postexposure was significantly higher after exposure to fine (β=2.53 mm Hg, *P*=0.001) and coarse (β=1.56 mm Hg, *P*=0.03) CAPs relative to measurements after the control exposure. Postexposure differences in diastolic BP were not statistically significant (Figure 2).

To confirm these findings, we also estimated the average within‐participant BP change in postexposure measurements relative to preexposure for each of the 3 exposures. Both systolic and diastolic BP showed significant average within‐participant postexposure increases after fine CAPs (β=2.97 mm Hg, *P*=0.002 for systolic BP; and β=1.87 mm Hg, *P*=0.005 for diastolic BP), and coarse CAPs (β=2.11 mm Hg, *P*=0.0008 for systolic BP; and β=2.36 mm Hg, *P*=0.0007 for diastolic BP). However, as expected due to normal circadian variations in BP, we also found in the control experiments a moderate postexposure increase in systolic BP (β=0.25 mm Hg, *P*=0.74) and a stronger increase in diastolic BP (β=1.83 mm Hg, *P*=0.006), albeit both were less pronounced than those found after fine or coarse CAPs exposures.

### Association of DNA Methylation With Blood Pressure

**Figure 3.**
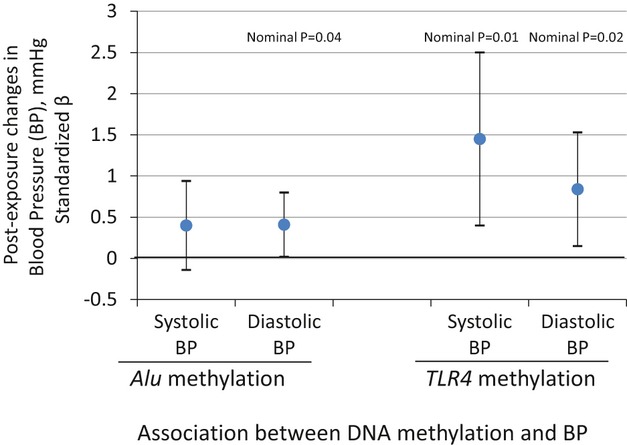
Associations of Alu and TLR4 methylation with systolic and diastolic blood pressure (BP). Standardized βs and 95% confidence intervals are shown in the figure. Standardize βs express the changes in BP as fractions of the BP standard deviation associated with a decrease in DNA methylation equal to its standard deviation. TLR4 indicates toll‐like receptor‐4.

To reduce false positive findings from multiple testing, we prioritized and present only the results on the association between DNA methylation and BP for the 2 markers that showed effects from CAP exposures, that is, *Alu* and *TLR4*. Decreased *Alu* methylation was associated with significantly increased diastolic BP (β=0.41, *P*=0.04) and nonsignificantly increased systolic BP (β=0.40, *P*=0.15). Decreased *TLR4* methylation was associated with significant increases of both diastolic (β=0.84, *P*=0.02) and systolic BP (β=1.45, *P*=0.01). Analyses stratified by exposure type showed correlations between DNA methylation and BP substantially homogeneous across the different exposures (data not shown) (Figure 3).

### Sensitivity Analyses

By study design, exposure‐order randomization is expected to balance potential confounders across exposure groups. We adjusted in our primary analysis for experiment order to limit confounding from random order imbalances. Moreover, by fitting a random intercept for each participant, our modeling approach allowed for controlling of participant characteristics that do not vary over time. A source of residual confounding may be represented by carryover effect. To control for this potential confounder we performed a sensitivity analysis adjusting models (1) and (2) for the previous exposure (binary variable indicating if the previous exposure was either CAPs or medical air). No major differences in the results were observed (Tables S6 and S7). As additional sensitivity analyses, multiple regression models were fitted to control for potential time‐varying confounders such as white blood cell counts and proportions of blood leukocyte cell types in differential blood counts (neutrophils, lymphocytes, monocytes, eosinophils, and basophils). We first checked if there was any difference in blood cell proportions between pre‐ and postexposure samples or when comparing exposure types in postexposure blood samples, and found no statistically significant differences (Table S8 and S9). Nonetheless, we further adjusted model (1) for white blood cell counts and proportions of blood leukocyte cell to exclude potential influences on DNA methylation. Results from these adjusted models (Table S10) were similar to those from our primary analysis.

### Mediation Analysis

We performed mediation analysis to estimate the proportion of the effects of the exposures on BP that were mediated by postexposure changes in DNA methylation. *Alu* methylation satisfied the underlying assumptions for mediation analysis, described in the methods section, as it showed significant associations with fine CAPs exposures (β=−0.74, adjusted *P* value=0.03), as well as with systolic BP in the model including both *Alu* methylation and fine CAPs exposure as independent predictors (β=−0.37, *P*=0.03). These results fulfill the assumptions for mediation analysis; therefore, we considered the potential pathway of fine CAPs exposure, *Alu* methylation and systolic BP and estimated indirect effect and proportion of mediation.

In the mediation analysis models, estimates of the proportion of mediation indicated that *Alu* hypomethylation mediated 10% of the positive association between fine CAP exposure and systolic BP (indirect effect: 0.40).

## Discussion

In the present study of controlled human exposures, fine and coarse CAPs induced blood hypomethylation of the *Alu* repetitive elements and *TLR4* gene. Hypomethylation of both *Alu* and *TLR4* was found to be associated with increased systolic BP after the exposures. A wealth of epidemiological studies have reported associations between peaks of ambient particulate matter exposure and cardiovascular disease and deaths.^4–5^ Several biological mechanisms that may in part explain these findings have been reported through the use of controlled human exposures to CAPs.^4^ Blood pressure, a well‐known predictor of cardiovascular risk, has been shown to exhibit small, but potentially critical acute increases in response to fine CAPs exposures.^9^ Decreased global and gene‐specific DNA methylation has been proposed as a novel mechanism mediating the effect of air pollution exposure to CVD‐related events.^19^ Castro et al^20^ found lower DNA methylation content in peripheral blood leukocytes from patients with atherosclerotic cardiovascular disease. Furthermore, recent findings from the Normative Aging Study have shown that lower blood LINE‐1 methylation predicts incidence and mortality from ischemic heart disease and stroke.^14^ Processes related to cardiovascular disease, such as oxidative stress,^21^ atherosclerosis,^20^ and aging^22^ have been associated with lower DNA methylation content in blood. In vascular tissues, hypomethylated DNA has been shown to be prone to mutations or aberrant gene expression patterns leading to the transition from a normal phenotype to vascular fibrocellular lesions by increasing proliferation of vascular smooth cells and lipid deposition.^20^

Previous in vitro experiments have shown that repetitive‐element and gene‐specific hypomethylation is induced by biological processes, such as oxidative stress,^21^ which are generated by PM in exposed individuals.^4^ Oxidative DNA damage can interfere with the ability of methyltransferases to interact with DNA,^21^ thus resulting in hypomethylation of cytosine residues at CpG sites. The association between ambient particle exposure and DNA methylation has been observed in repetitive elements, such as *Alu* and LINE‐1,^15^ in innate immune and proinflammatory pathways (*TLR4*,* IL‐12*,* IL‐6*),^17–18^ and in the inducible nitric oxide synthase gene (*iNOS*).^16^

In our study, *Alu* and *TLR4* methylation were found to be significantly lower after CAPs exposures, providing for the first time—to the best of our knowledge—direct experimental evidence that PM exposure induces DNA hypomethylation in humans. We also observed a significant increase in systolic BP after CAPs exposures, confirming previous results on fine particle exposure^4^ and providing novel experimental evidence indicating that coarse exposure induces cardiovascular responses. Our findings also provide results consistent with the hypothesis that rapid hypomethylation of *Alu* and *TLR4* is an epigenetic mechanism that mediates the effects of particle exposure on BP. Activation of *Alu* repetitive elements, which is associated with hypomethylation of genomic DNA, increases in older individuals and has been suggested to contribute to biological aging and age‐related chronic disease.^33^ Nearly 1 million copies of *Alu* sequences are dispersed throughout the genome.^34^ *Alu* methylation states have been shown to control DNA compaction and alter the expression of nearby genes.^34^ Previous studies have linked the presence of *Alu* sequences in a number of genes related with hypertension—including the serine/threonine protein kinase family member *WNK1*,^35^ angiotensin I converting enzyme,^36–37^ tissue‐type plasminogen activator,^38^ and 11beta‐hydroxysteroid dehydrogenase type 2.^39^ Whether the association found in our study between global *Alu* hypomethylation and BP is driven by hypomethylation of specific *Alu* sequences in these genes, or in other genomic regions, remains to be determined.

Recent investigations have consistently indicated that expression of *TLR4* on circulating leukocytes may act as a primary communication between the innate immune system and systemic vascular functions. Experimental models have shown that *TLR4*, which is activated by endotoxin contained in PM, contributes to induce PM‐related inflammation, oxidative stress, and CVD‐related responses.^40–41^ Kampfrath et al^40^ showed that TLR4 deficiency prevented the increased microvascular vasoconstriction induced in mice by PM_2.5_ exposure, as well as PM‐related inflammatory and oxidative‐stress responses. Bonfim et al^41^ showed that TLR4 inactivation with anti‐TLR4 antibodies reduced the mean arterial pressure in spontaneously hypertensive rats. Recent molecular studies have shown that TLR4 binds a wide range of endogenous ligands related to BP regulation and cardiovascular function, including angiotensin II (AT‐II), heat shock protein, fibrinogen, and fibronectin.^41^ Downstream products of TLR4 signaling include thromboxane A2, a potent vasoconstrictor that can induce rapid increases in BP.^42^ TLR4 expression is increased in both peripheral mononuclear leukocytes and cardiac tissues of hypertensive patients.^25,43^ Our results are consistent with these animal models that indicated PM‐induced activation of TLR4 pathways as integral to PM‐related cardiovascular effects.

Results from regression models in the present study were reported as standardized beta that indicated the effects of the exposures as a fraction of a SD of the DNA methylation markers. This presentation allowed to compare the size of CAPs effects across different markers and also showed that the effects of CAPs on DNA methylation were sizable relative to the methylation SDs, as—for instance—they amounted to as much as 74% of the SD of *Alu* methylation. However, the SDs of both *Alu* and *TLR4* were small, thus indicating that *Alu* and *TLR4* methylation is tightly regulated. These findings suggest that even small changes in absolute DNA methylation, potentially corresponding to profound demethylation in a subset of blood leukocytes, may lead to effects on BP. In our study, CAP exposures did not show consistent effects on LINE‐1 methylation. Methylation measures in LINE‐1 and *Alu* repetitive elements have been proposed as markers of genomic DNA methylation content based on results in cancer cells.^34^ However, the 2 repetitive elements are controlled through different mechanisms and have been shown to have different transcription patterns in response to environmental stressors and other conditions.^33^ For instance, *Alu*—but not LINE‐1—methylation has been shown to decrease through aging,^33^ potentially reflecting differential susceptibility to cumulative environmental insults over time. Our data provide further evidence that *Alu* and LINE‐1 methylation have different sensitivities to environmental stressors,^15^ and that they can show different associations with cardiovascular outcomes, such as increased BP. In contrast to previous evidence from in vitro and observational investigations,^16–18^ CAPs exposures did not affect *IL‐6*,* IL‐12*, or *iNOS* methylation in our study. Despite the advantage of using high‐precision quantitative pyrosequencing measures, the lower number of CpG sites analyzed in these genes, compared to those measured in *Alu* and *TLR4*, may have limited our capability to detect potential effects. Blood DNA is derived from a mixed cell population of different types of circulating white blood cells. Therefore, our findings on DNA methylation may have resulted from a CAP‐induced shift in cell populations. However, CAPs exposure had no significant effects on the proportions of the major leukocyte cell types and sensitivity analysis showed no substantial differences in the results from models adjusted for leukocyte total count and proportions of neutrophils, lymphocytes, monocytes, basophils, and eosinophils. Although these findings limit the chances that shifts on major cell types underlie CAP‐induced effects on DNA methylation, it is still possible that our results may be driven by changes in other smaller subpopulations, such as the different types of circulating lymphocytes. TLR4 has been found to be expressed in a variety of hematopoietic cells, including lymphocytes, monocytes, and myeloid cells.^24^ Future studies are needed to confirm our findings and determine the cell compartment responsible for the observed changes. Finally, the volunteers enrolled in the present study were healthy and not necessarily representative of the general population. Our results may not be generalizable to different population strata and, particularly, should be replicated in a higher risk population. Moreover, the relatively small sample size might have limited our capability to detect significant effects.

In conclusion, our results demonstrate for the first time in a human experimental study that PM exposure induces rapid DNA hypomethylation. *Alu* and *TLR4* hypomethylation may represent a novel mechanism that mediates environmental effects on BP.
